# Effects of Chronic Barley Consumption on Upper Respiratory Tract Symptoms in Japanese Healthy Adults: A Randomized, Parallel-Group, Controlled Trial

**DOI:** 10.3390/nu16142298

**Published:** 2024-07-17

**Authors:** Risa Araki, Chiaki Ishikawa, Tomomi Kawasaki, Toshiro Kobori, Toshihiko Shoji, Yoshiharu Takayama

**Affiliations:** Division of Food Function Research, Institute of Food Research, National Agriculture and Food Research Organization (NARO), 2-1-12 Kannondai, Tsukuba 305-8642, Ibaraki, Japan; ishikawac956@affrc.go.jp (C.I.); kawasakit689@affrc.go.jp (T.K.); tkobo@affrc.go.jp (T.K.); tshoji@affrc.go.jp (T.S.); takay@affrc.go.jp (Y.T.)

**Keywords:** barley β-glucan, Profile of Mood States (POMS), natural killer (NK) cells, upper respiratory tract symptoms (URTS), waxy barley, Wisconsin Upper Respiratory Symptom Survey (WURSS)-21

## Abstract

β-(1,3/1,4)-glucan is a major component of cereal grains, such as oats and barley. In this study, we investigated the effects of cooked waxy barley, which contains β-(1,3/1,4)-glucan, on upper respiratory tract physical symptoms and mood status by performing a randomized, parallel-group, comparative trial. The primary outcome was assessed using the Wisconsin Upper Respiratory Symptom Survey-21 and Profile of Mood States second edition. Twenty-seven healthy Japanese adult participants were supplemented with 100 g of cooked waxy barley (containing 1.8 g of β-glucan) or 100 g of cooked white rice daily for 8 weeks. Participants receiving cooked waxy barley reported a reduction in cumulative days of sneezing (*p* < 0.05) and feeling tired (*p* < 0.0001) compared with the control group. After the intervention period, there were significantly less severe nasal symptoms, such as runny nose, plugged nose, and sneezing (*p* < 0.05), and a significantly greater reduction of the Tension-Anguish score (*p* < 0.05) in the barley group than in the control group. This study suggests that supplementation of cooked waxy barley containing β-(1,3/1,4)-glucan prevents or alleviates nasal upper respiratory tract symptoms and improves mood status. The findings of this study should be confirmed by double-blind trials with a larger number of participants.

## 1. Introduction

According to numerous systematic reviews and meta-analyses, consumption of fiber-rich foods significantly reduces the risk of lifestyle diseases such as obesity and diabetes, as well as coronary heart disease [1,2]. β-D-glucan (β-glucan) is a dietary fiber found in yeast, seaweed, mushrooms, wheat, and barley. It consists of D-glucose polymerized into linear or branched chains linked by β-glycoside linkages [3]. β-glucan is an indigestible dietary fiber that is not enzymatically digested in the stomach and reaches the small intestine. An aqueous solution of β-glucan has a high viscosity, which prolongs its retention time in the intestine, thereby lowering the absorption rates of lipids and carbohydrates [3,4]. β-glucan can bind to bile acids and affects bile acid and lipid metabolism by accelerating fecal bile acid excretion, accompanied by decreased plasma bile acid levels [3,4,5]. In addition, numerous studies have indicated that β-glucan consumption lowers postprandial blood glucose levels and antagonizes high-fat diet-induced elevation of blood low-density lipoprotein cholesterol levels, leading to a reduced risk of cardiovascular disease and diabetes [6,7,8,9].

β-glucans are heterogeneous glucose polymers that differ in terms of the orientation of glycosidic linkages, chain length, and degree of branching, resulting in diverse physiological properties [3,4]. Cereals, such as barley and oats, are important sources of dietary fiber. β-(1,3/1,4)-glucan is a major component of cereal granules [3]. The primary structure of cereal-derived β-(1,3/1,4)-glucan is a linear polymer of β-(1,4) cellotriose or β-(1,4) cellotetraose units linked by single β-(1,3) glycosidic linkages [4]. The clinical benefits of oat-derived water-soluble β-glucan, such as reduction of plasma cholesterol levels and the risk of coronary heart disease, have been confirmed by The United States Department of Agriculture and European Food Safety Authority [10,11].

On the other hand, β-glucan found in yeast and fungi has a β-(1,3)-linked glucan backbone, which is branched with β-(1,6) linkages (β-(1,3/1,6)-glucan) [3]. Orally administered β-(1,3/1,6)-glucan can reach the intestinal tract and activate the innate immune system [12]. β-(1,3/1,6)-glucan is a pathogen-associated molecular pattern that activates numerous pattern recognition receptors, including dectin-1, Toll-like receptor-2, and complement receptor 3 (CR3) [12,13,14]. β-glucans recognized by pattern recognition receptors trigger innate immune responses mediated by host immune cells. For example, β-glucans activate macrophages by binding to dectin-1 concomitant with Toll-like receptor-2 [12,14]. In phagocytes and natural killer (NK) cells, the binding of β-glucan fragments to CR3 elicits cytotoxicity against iC3b-opsonized tumor target cells [13,14,15]. A well-known immunomodulatory β-glucan is Zymosan, a mixture of mannan and β-glucan extracted from yeast cell walls [3,12,15]. Zymosan facilitates phagocytosis of pathogens by polymorphonuclear leukocytes, macrophages, and other phagocytic cells [15]. Therefore, Zymosan is a commonly used adjuvant as an activator of the alternative complement pathway. Another well-characterized immunomodulatory β-glucan is lentinan, which is purified from the fruiting body of shiitake mushroom (*Lentinula edodes*) [3,12,14,15]. The inhibitory effect of lentinan (or lentinan-based chemical drugs) on tumor growth has been confirmed in both clinical and animal studies. Lentinan exerts its anticancer property by activating NK cells or promoting lymphocyte proliferation [14]. 

Upper respiratory tract symptoms (URTS) are common and mainly caused by infection of respiratory viruses [16]. In addition, increased exposure to environmental allergens, such as pollens, mites, and dust, may cause asthma or allergies, leading to the onset of non-infectious URTS [16]. In general, URTS are characterized by increased nasal symptoms, such as runny nose, plugged nose, and repetitive sneezing, as well as respiratory and mucosal symptoms [16]. Improvement of immune function may reduce the incidence of both infectious and non-infectious URTS [16]. 

Previous intervention studies have reported that yeast-derived β-(1,3/1,6) glucans reduce the severity or duration of URTS, mainly in participants who perform intensive physical activity or precipitants with a tendency to catch colds frequently [17,18,19,20,21]. The beneficial effect of β-glucan consumption is likely due to the broad physiological activities of β-glucans, including immunomodulatory and antioxidant activities. Similar results have been obtained with β-(1,3) glucan extracted from *Euglena gracilis* [22,23]. The participants received β-(1,3) glucan derived from *Agrobacterium* or β-(1,3/1,6) glucan derived from *Ganoderma lucidum* (Reishi mushroom) showed higher NK cell activity compared to control groups [24,25]. By contrast, the immunomodulatory effect of cereal-derived β-(1,3/1,4)-glucan has only been demonstrated in animal studies [26]. Little is known about the immunomodulatory effects of cereal-derived β-(1,3/1,4)-glucan in humans. The purpose of this study was to evaluate the immunomodulatory effects of cereal-derived β-glucan by performing an intervention trial. We investigated whether continuous consumption of 100 g of cooked waxy barley once a day for 8 weeks was more effective than consumption of the same amount of cooked white rice in improving subjective physical symptoms related to URTS and mood states, using the Wisconsin Upper Respiratory Symptom Survey (WURSS)-21 [27] and the Profile of Mood States second edition (POMS 2) [28].

## 2. Materials and Methods

### 2.1. Study Design

An investigator-blind, placebo-controlled, parallel-group study was conducted from 25 January to 26 April 2023, by research staff at the Institute of Food Research, National Agriculture and Food Research Organization (NARO). 

Healthy Japanese adults who usually consume cooked white rice as a staple food at least once per day were recruited from panelists lived in Tokyo metropolitan area and registered with imeQ RD Inc. (Tokyo, Japan), the Foods with Health Claims development support business (Food CRO), or employees working in NARO’s Tsukuba region as candidates for this study. They were recruited by the research manager of imeQ RD inc. or Institute of Food Research, NARO, through email or online message boards from 14 December 2022 to 28 February 2023.

**Figure 1:**
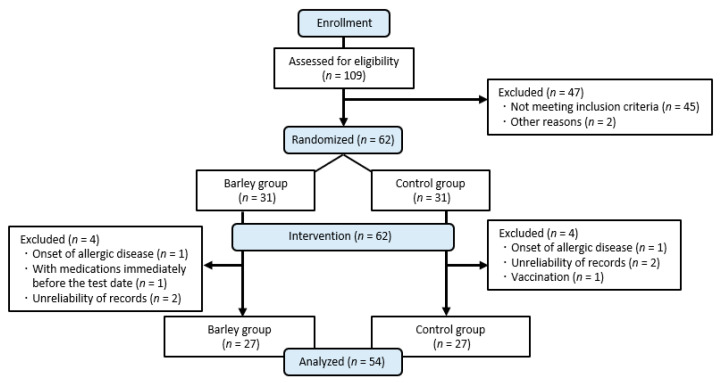
Diagram illustrating the selection of participants for analysis.

Candidates who (1) were currently being treated for chronic diseases, (2) had a current or past history of any drug or food allergy, (3) regularly used dietary supplements that affect immune function, (4) were shift workers, (5) were heavy smokers, (6) were heavy drinkers, (7) were enrolled or planned to enroll in other clinical trials within 1 month during the period of our study, and (8) were deemed unsuitable for this study by the principal investigator were excluded. A total of 109 candidates were screened for eligibility from those who attended the information session and provided written consent (Figure 1). Then, 62 eligible participants were selected based on the results of biochemical tests by mail using a novel capillary blood sampling device [29], and background information was obtained from a questionnaire. Once enrolled as intervention participants, they were randomly assigned to the control and barley groups (31 in each group) by the data coordinator. Simple randomization using a computer-generated sequence of numbers was used for allocation. The allocation sequence was concealed from research staff during the intervention. After the data were fixed, the allocation was disclosed to research staff, including the data analyst. In total, 8 participants who deviated from the protocol during the intervention period were excluded, and 54 participants (27 in each group) were finally included in the analysis.

This clinical trial has been registered in the University Hospital Medical Information Network (UMIN, http://www.umin.ac.jp/ctr accessed on 16 December 2022; UMIN000049817). The data were collected at the Nishi-Tokyo Sakura Clinic (Tokyo, Japan) or the Institute of Food Research, NARO (Ibaraki, Japan), between 25 January and 26 April 2023, by research staff of the Institute of Food Research, NARO.

### 2.2. Test Foods

The control group was given packed cooked white rice (TableMark. Co., Ltd., Tokyo, Japan), and the barley group was given packed cooked waxy barley “Sanuki Mochi Wheat Daishi Mochi” (Mandegan Co., Ltd., Kagawa, Japan) with a daily serving size of 1 package (100 g) for both groups. Daishimochi is a six-row naked barley variety bred at the Shikoku Agricultural Experiment Station of the Ministry of Agriculture, Forestry and Fisheries (currently known as the West Japan Agricultural Research Centre, NARO, Fukuyama, Hiroshima, Japan) and was registered as a variety in 2000 [30,31]. Its seedlings contain about 6% β-glucan. This is 1.5 times the average β-glucan content (4.1%) of 30 barley and naked barley varieties [32]. The nutritional composition of each test food is shown in Table 1. In appearance, cooked waxy barley for the barley group was black-purple because it contained anthocyanins [30], while cooked white rice for the control group was milky white.

Participants were required to (1) heat the test foods in a microwave oven prior to consumption; (2) if their usual rice intake per meal prior to the study exceeded 100 g, consume additional cooked rice with the test food and adjust their total daily intake of staple foods during the intervention period to approximately the same amount as that prior to the study; and (3) measure their intake of test foods and other staples on a kitchen scale and report daily online via digital diary.

### 2.3. Outcomes

Questionnaire-based physical conditions and mood status scores were used as the primary outcome. Daily subjective physical conditions were assessed using a WURSS-21-based questionnaire [27]. Participants were asked to report the severity of each of 10 symptoms associated with URTS daily throughout the 8-week intervention period using an online visual analogue scale in the digital diary, ranging from 0 (no symptoms) to 7 (severe). Scores were recorded to one decimal place. For each symptom, a score of 5 or higher was treated as symptom-positive. The cumulative number of days participants were symptom-positive during the intervention was counted for each group. Similarly, patients were asked to report online daily their waking body temperature, whether and how much of the test food they had consumed, and any special notes. The cumulative number of days participants had a fever of 37.5 °C or higher, took medication, or visited a medical facility was also counted. In addition, the mean scores were calculated for week 1, week 8, and amount of change by subtracting week 8 from week 1. Participants’ mood states over the previous 7 days, including the day of measurement, were assessed twice, at baseline and the end of the study. These were assessed using the POMS 2—Adult Short Version [28], which consists of 35 questions on a five-point scale, ranging from 0 (not at all) to 4 (extremely). Raw scores for the total mood disturbance (TMD) and seven subscales, namely Anger-Hostility (A-H), Confusion-Bafflement (C-B), Depression-Dejection (D-D), Fatigue-Intolerance (F-I), Tension-Anguish (T-A), Vigor-Activity (V-A), and Friendliness (F), were calculated from the survey responses. The TMD score was calculated by subtracting V-A from the sum of A-H, C-B, D-D, F-I, and T-A. These were then converted into a T-score, which was standardized based on age and sex. 

NK cell activity was a secondary outcome. Venous heparinized blood samples for analysis by the ^51^Cr-release assay according to the method of Elsner et al. [33] at Kotobiken Medical Laboratories Inc. (Ibaraki, Japan) were collected at baseline and the end of the study after an overnight fast of at least 12 h. Blood sampling was performed by qualified medical professionals at the Nishi-Tokyo Sakura Clinic (Tokyo, Japan) or the Institute of Food Research, NARO (Ibaraki, Japan). 

### 2.4. Statistical Analyses

A priori sample size calculation could not be performed because this was an exploratory study, and no reference data were available. However, a post hoc power analysis using G*Power 3 [34] showed that a sample size of 27 participants per group gave a power of 72%, 39%, 67%, and 82% to detect between-group differences in changes in poor health, runny nose, plugged nose, and sneezing scores during the intervention period, using a Mann–Whitney U test with a two-sided significance level of *p* < 0.05. 

Descriptive statistics are presented as median with interquartile range except if stated otherwise. The Shapiro–Wilk test and graphs (Q-Q plots and histograms) were used to test the normality assumption for quantitative variables. The Mann–Whitney U test was used to compare the mean values of each parameter between the two groups. Within-group differences were compared using the Wilcoxon signed-rank test. To compare the frequency distribution of categorical data, the Chi-square test was used.

The level of statistical significance was defined as a *p*-value less than 0.05 using a two-tailed test. IBM SPSS Statistics 26 (IBM Japan, Ltd., Tokyo, Japan) was used for all statistical analyses.

## 3. Results

### 3.1. Characteristics of Participants

Of the total intervention population, 35.2% were men, and 64.8% were women. Their age and body mass index at enrolment were 46 (33–50) years and 20.6 (19.4–22.4) kg/m^2^, respectively. Baseline characteristics and the rate of completion of the test food during the intervention did not significantly differ between the two groups (Table 2).

### 3.2. Physical Conditions

#### 3.2.1. Cumulative Days with URTS-Related Symptoms and Special Notes

The cumulative prevalence of each symptom in the whole study population was only 0.1–2.1%, and all symptoms disappeared within a few days. No adverse events were observed that would suggest a causal relationship with the test food.

The numbers of cumulative days of sneezing (*p* < 0.05) and feeling tired (*p* < 0.0001) were lower in the barley group than in the control group (Table 3). In terms of the cumulative number of days of special notes, the number of cumulative days of taking medication was lower in the barley group than in the control group (*p* < 0.05, Table S1).

#### 3.2.2. Severity of URTS-Related Symptoms

**Figure 2:**
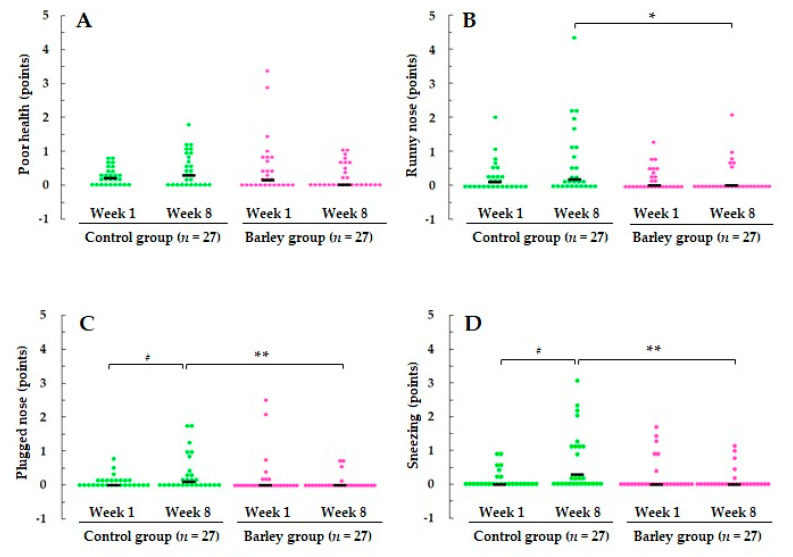
Scores for (**A**) poor health, (**B**) runny nose, (**C**) plugged nose, and (**D**) sneezing at weeks 1 and 8. The scores for each individual participant are plotted on the graph, and the median score is indicated by a horizontal bar.* *p* < 0.05, ** *p* < 0.01 vs control group based on the using the Mann–Whitney U test. ^#^ *p* < 0.05 vs. week 1 based on the Wilcoxon signed-rank test. Severity of symptoms was rated on an eight-point scale: 0 = no symptoms, 1 = very mild, 3 = mild, 5 = moderate, and 7 = severe. URTS, upper respiratory tract symptoms.

No symptom score significantly differed between the two groups at week 1 (Figure 2, Table S2). In the control group, there was a significant worsening of plugged nose and sneezing (both *p* < 0.05) and a trend toward a worsening of poor health (*p* = 0.052) and runny nose (*p* = 0.055) at week 8 compared with week 1. By contrast, the barley group showed no change in these symptom severities between the two time points and had significantly better scores for runny nose (*p* < 0.05), plugged nose, and sneezing (both *p* < 0.01) at week 8 than the control group (Figure 2).

**Figure 3:**
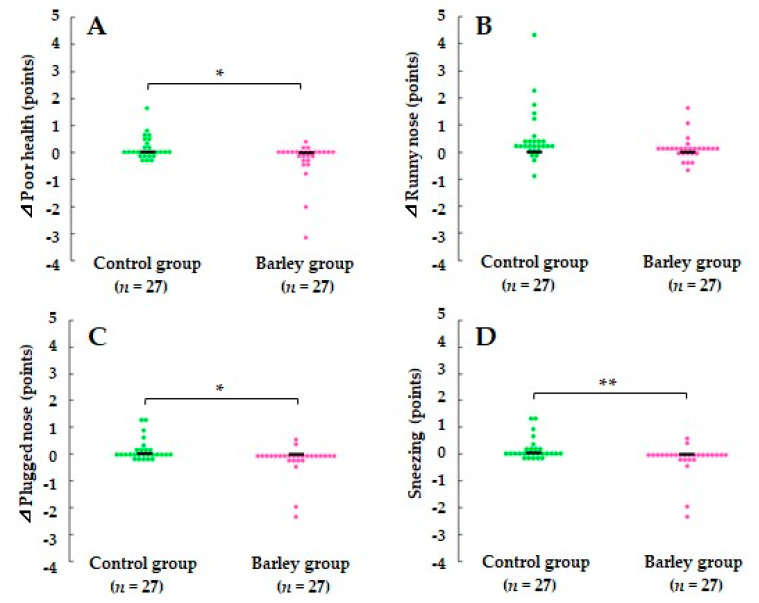
Comparison of changes in scores for (**A**) poor health, (**B**) runny nose, (**C**) plugged nose, and (**D**)sneezing between the two groups during the intervention period. The scores for each individual participant are plotted on the graph, and the median score is indicated by a horizontal bar. * *p* < 0.05 and ** *p* < 0.01 vs. the control group based on the Mann–Whitney U test.

During the intervention, scores for poor health, plugged nose (both *p* < 0.05), and sneezing (*p* < 0.01) were significantly reduced in the barley group compared with the control group. In addition, there was a tendency for the increase of the runny nose score to be smaller in the barley group than in the control group (*p* = 0.073) (Figure 3). Although other symptoms did not appear to substantially differ between the two groups, scores for all symptoms were reduced in the barley group (Figure S1).

### 3.3. Mood Status

**Figure 4:**
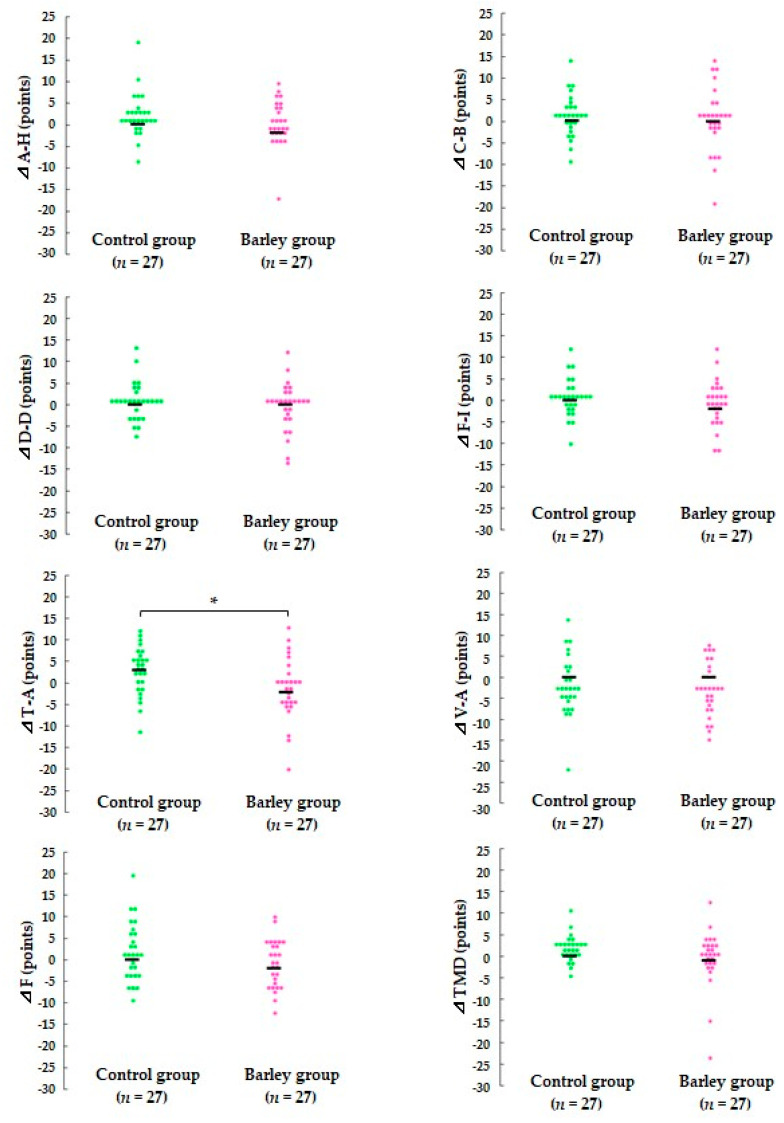
Comparison of changes in scores for POMS2 T-score between the two groups during the intervention period. The scores for each individual participant are plotted on the graph, and the median score is indicated by a horizontal bar. * *p* < 0.05 vs. the control group based on the Mann–Whitney U test. A-H, Anger-Hostility; C-B, Confusion-Bewilderment; D-D, Depression-Dejection; F-I, Fatigue-Inertia; T-A, Tension-Anxiety; V-A, Vigor-Activity; F, Friendliness; TMD, total mood disturbance. The TMD score was calculated by subtracting V-A from the sum of A-H, C-B, D-D, F-I, and T-A.

After the intervention, T-A scores significantly increased in the control group (*p* < 0.05), and there were decreasing trends in F-I (*p* = 0.083) and F (*p* = 0.060) scores in the bar-ley group (Table S3). The barley group also had negative changes in other negative mood subscales (A-H, C-B, D-D, and T-A scores). Among these, a greater reduction in the T-A score was observed in the barley group (*p* < 0.05) than in the control group, and the TMD score (*p* = 0.096) also showed a tendency to decrease (Figure 4).

### 3.4. NK Cell Activity

**Figure 5:**
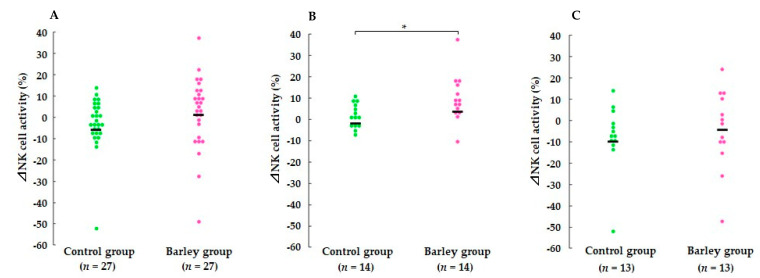
Comparison of changes in NK cell activity between the two groups during the intervention period in (**A**) all participants, (**B**) the low initial NK cell activity group, and (**C**) the high initial NK cell activity group. The values for each individual participant are plotted on the graph, and the median value is indicated by a horizontal bar. * *p* < 0.05 vs. the control group based on the Mann–Whitney U test.

In the whole study population, NK cell activity in the control group was significantly lower at week 8 than at week 1 (*p* < 0.01), and the reduction tended to be greater than in the barley group (*p* = 0.085). We used a stratified subgroup analysis approach to compare the effect of barley consumption on NK cell activation according to their baseline activity. Median NK cell activity at week 1 (36%) was used as a cutoff to divide the target into two groups. In the high baseline NK cell activity group, NK cell activity did not differ within the same group or between groups at any time point. Meanwhile, in the low baseline NK cell activity group, NK cell activity was significantly increased by the intervention, and the extent of the elevation was significantly greater in the barley group than in the control group (both *p* < 0.05) (Figure 5, Table S4).

## 4. Discussion

To our knowledge, this is the first randomized, parallel-group, comparative study to report that cooked waxy barley intake attenuates the severity and duration of upper respiratory tract infection (URTI) episodes in healthy adults. Among 11 subjective physical symptoms in WURSS-21, poor health and three nasal symptoms were significantly improved in the barley group compared with the control group (Figure 2 and Figure 3). In addition to attenuation of URTS episodes, participants receiving cooked waxy barley showed an improved mood status compared with those in the control group, as assessed by the POMS 2 questionnaire. The T-A score was reduced more during the intervention period in the barley group than in the control group (*p* < 0.05) (Figure 4). 

### 4.1. Scientific Significance of This Study 

The present study builds upon previous findings showing that yeast-derived β-glucan (β-(1,3/1,6)-glucan) and Euglena-derived β-glucan (β-(1,3)-glucan) alleviate the development of URTS episodes in participants who perform exercise vigorously or are sensitive to cold [17,18,19,20,21]. The subjects of human studies showing the beneficial effects of β-glucan consumption were not restricted to a particular race or ethnicity [17,18,19,20,21]. Therefore, the effects of β-(1,3/1,6)-glucan appear to be independent of the genetic backgrounds of the participants. It is generally accepted that a high URTS incidence episode is associated with intense exercise events, such as marathon running, and is most often characterized by attenuated innate immune function. Increased susceptibility to URTS correlates with the downregulation of NK cell activity [35] and elevation of pro-inflammatory cytokines [36]. NK cells are an important member of the innate immune system due to their antiviral function. Although the nature of NK cells in the upper respiratory tract is not well characterized, the number of these cells in the upper respiratory tract increases in response to viral infection [37]. In this study, we found that waxy barley intervention affected NK cell activity (Figure 5, Table S4). The effects of barley intervention were relatively apparent in participants with low baseline NK cell activity, but not statistically significant in those with high baseline NK cell activity (Figure 5, Table S4). We speculate that the effect of barley consumption was limited to the population with low baseline NK cell activity. 

The immunomodulatory effects of β-(1,3/1,4)-glucan have been suggested by previous in vitro and in vivo studies. Davis et al. reported that increased morbidity in HSV-1 infected CD-1 mice due to exercise stress was diminished by the feeding of oat-derived β-glucan [38]. Oat-derived β-glucan attenuated the reduction of antiviral resistance in macrophages, suggesting that β-glucan ameliorates the exercise-induced URTI risk [38]. Estrada et al. reported that oat-derived β-(1,3/1,4)-glucan stimulated the production of inflammatory cytokines by macrophages in vitro [39]. They also reported that intraperitoneal administration of β-(1,3/1,4)-glucan in mice resulted in accumulation of macrophages in the peritoneal cavity [39]. Therefore, β-(1,3/1,4)-glucan is proposed to reduce the incidence of URTIs by regulating the innate immune system. However, the effects of various β-(1,3/1,4)-glucan intervention studies on plasma inflammatory cytokines (IL-6, IL-18 and C-reactive protein) have been controversial [26]. Few interventional studies have demonstrated the immunomodulatory function of β-(1,3/1,4)-glucan. Our observations in this intervention trial are consistent with the previous finding that intake of barley-derived carbohydrate powder relieves symptoms of nasal allergy, such as sneezing, runny nose, and congestion [40]. Further study is required for better understanding the property of β-(1,3/1,4)-glucan on innate immune systems.

Another well-known immunomodulatory compound found in Daishimochi is anthocyanin [41]. Anthocyanin is a purple flavonoid pigment concentrated in bran of the grain of certain types of barley and wheat [42]. Among several types of anthocyanin, cyanidin 3-*O*-(3,6-di-O-malonyl-β-d-glucopyranoside) is the main anthocyanin detected in Daishimochi [43]. Anthocyanin has immunomodulatory activity by reducing oxidative stress [41]. It has been reported that the total mean content of anthocyanin in the grain of Daishimochi is less than 10 mg/100 g [43]. In addition, about half of anthocyanin in grain is discarded during the peeling process after harvest [43]. In human intervention trials to verify the immunostimulatory effect of anthocyanins, anthocyanin dosages have generally been set at 250–300 mg or higher per day [42], and the amount of anthocyanins from Daishimochi intake in our intervention study is insufficient to exert an immunomodulatory effect.

Numerous studies have sought to elucidate the mechanisms by which β-glucans regulate the innate immune system. Microfold cells are a unique subset of intestinal epithelial cells in Payer’s patches for transepithelial transport of macromolecules [44]. They are considered the initial site of mucosal immunity against pathogens or exogenous antigens, including β-glucans [3]. β-glucans are recognized by receptors on the host’s immune cells. 

### 4.2. Mechanism Underlying the Immunomodulatory Effect of β-(1,3/1,4) Glucans 

Previous in vitro studies have shown that β-glucan derived from mushrooms or yeast activates various immune cells, such as macrophages, dendritic cells, and NK cells, by binding to dectin-1 or CR3, respectively [12]. The β-(1,3)-glucan backbone was thought to be responsible for binding of β-glucan to dectin-1 [12]. Meanwhile, only a few studies have investigated the immunomodulatory mechanisms of cereal-derived β-(1,3/1,4)-glucan. β-glucans derived from oats and barley lack the β-(1,6)-linkage, and their affinity for dectin-1 is lower than that of β-glucans derived from fungi and yeast [45]. Therefore, we speculate that activation of NK cells in the barley group was mediated by a receptor(s) other than dectin-1. The mechanisms by which β-glucans prevent or alleviate nasal URTS remain to be elucidated.

Another mechanism underlying the immunomodulatory effect of β-glucans is likely indirect, depending on their role as prebiotics [3]. In the gastrointestinal tract, β-glucans are processed via fermentation by gut commensal bacteria, resulting in the production of short-chain fatty acids (SCFAs) [3,4]. SCFAs antagonize fatty acid accumulation in adipocytes by activating the G-protein-coupled receptor (GPR) 41- and GPR43-mediated signaling pathway [3,15]. β-glucans induce a shift in the population of the intestinal microbiota and SCFA profiles due to fermentation by gut microbiota [3]. Many immune cells, including neutrophils, macrophages, dendritic cells, mast cells, and lymphocytes, express GPR43 and GPR109a (also known as HCR2), which are involved in regulating inflammatory responses, suggesting that β-glucans exert their biological activity by regulating gut microbiota [3,46,47].

In this study, participants were given cooked waxy barley containing approximately 1.8 g of β-(1,3/1,4)-glucan per day. Previous interventions aimed at reducing URTI episodes used less than 1.0 g of β-(1,3/1,6)-glucan per day [17,18,19,20,21]. The differences in the orientation of glycosidic linkages, chain length, and degree of branching between β-glucans from different sources might affect their receptor affinity and biological properties. A further study is needed to optimize the dosage of β-(1,3/1,4)-glucans administered during the intervention period.

### 4.3. Limitations 

This study has some limitations. First, it was not double-blind because the appearance of the test food (Daishimochi cooked waxy barley) was different from that of the control food (cooked white rice) due to the purple pigment (anthocyanin) in the former. However, all measurements and data analyses were performed in a blind manner. Second, it was a non-crossover trial. Therefore, we cannot completely rule out the possibility that personal variation in the efficacy of β-glucan led to differences in the intervention effects between the two groups. Third, the effect of β-(1,3/1,4)-glucan may vary depending on the race and ethnicity of the participants. The participants of this study were residents of a specific area (greater Tokyo and others metropolitan area, Japan), and the number was relatively small, so future studies are needed to confirm generalizability. Fourth, it did not evaluate the effects of β-glucan intervention on production of pro-inflammatory cytokines, making it difficult to elucidate the mechanisms by which β-glucan affects innate immune cells.

## 5. Conclusions

Continuous intake of waxy barley may prevent or alleviate nasal URTI symptoms and improve mood status compared to white rice. Although the mechanism and functional component have not been fully identified, β-(1,3/1,4)-glucans may be responsible for most of the beneficial effects of waxy barley. Future research should aim to confirm these results in double-blind trials with larger number of participants. If our findings are generalized, promoting the consumption of waxy barley may contribute to improving the quality of life of healthy adults by preventing infectious diseases and psychological stress.

## Figures and Tables

**Table 1:** Nutritional composition of 100 g of each test food.

	Packed Cooked White Rice	Packed Cooked Barley
Energy (kcal)	147	117
Protein (g)	2.2	2.8
Fat (g)	0.2	1.0
Carbohydrate (g)	34.1	27.2
β-glucan (g)	0.0	1.8

**Table 2:** Characteristics of participants.

	Control Group (*n* = 27)		Barley Group (*n* = 27)		*p* ^b^
Male/Female (n)		10/17		9/18	0.776 ^c^
Age (years)	48.0	(33.0–55.0) ^a^	45.0	(30.0–52.0)	0.436
Body mass index (kg/m^2^)	20.7	(19.7–21.2)	20.5	(19.3–22.5)	0.945
The rate of completion of the test food (%)	100.0	(98.2–100)	100.0	(98.2–100)	0.906

^a^ Median with interquartile range. *p*-values were calculated using the ^b^ Mann–Whitney U and ^c^ Chi-square tests.

**Table 3:** Comparison of cumulative days with URTS-related symptoms between the two groups.

		Symptom		*p* ^a^
		Positive	Negative	
Fever	Control group (*n* = 27)	1	1507	0.375 ^b^
(days)	Barley group (*n* = 27)	3	1504	
Poor health	Control group (*n* = 27)	8	1500	0.489
(days)	Barley group (*n* = 27)	11	1496	
Runny nose	Control group (*n* = 27)	14	1494	0.296
(days)	Barley group (*n* = 27)	9	1498	
Plugged nose	Control group (*n* = 27)	10	1498	0.179
(days)	Barley group (*n* = 27)	7	1500	
Sneezing	Control group (*n* = 27)	10	1498	0.038 ^b^
(days)	Barley group (*n* = 27)	2	1505	
Sore throat	Control group (*n* = 27)	7	1501	0.795
(days)	Barley group (*n* = 27)	8	1499	
Scratchy throat	Control group (*n* = 27)	4	1504	0.266 ^b^
(days)	Barley group (*n* = 27)	8	1499	
Cough	Control group (*n* = 27)	6	1502	0.315
(days)	Barley group (*n* = 27)	10	1497	
Hoarseness	Control group (*n* = 27)	4	1504	0.687 ^b^
(days)	Barley group (*n* = 27)	2	1505	
Head congestion	Control group (*n* = 27)	7	1501	0.795
(days)	Barley group (*n* = 27)	8	1499	
Chest congestion	Control group (*n* = 27)	2	1506	1.000 ^b^
(days)	Barley group (*n* = 27)	1	1506	
Feeling tired	Control group (*n* = 27)	52	1456	<0.0001
(days)	Barley group (*n* = 27)	13	1494	

*p*-values were calculated using the ^a^ Chi-square and ^b^ Fisher’s exact tests. The number of cumulative days was 1508 (22 participants × 56 days, 3 participants × 55 days, 1 participant × 54 days, and 1 participant × 57 days) in the control group and 1507 (23 participants × 56 days, 3 participants × 55 days, and 1 participant × 54 days) in the barley group. URTS, upper respiratory tract symptoms.

## Data Availability

The raw data supporting the conclusions of this article will be made available by the authors on request.

## Supplementary Materials

The following supporting information can be downloaded at https://www.mdpi.com/article/10.3390/nu16142298/s1, Figure S1: Comparison of changes in scores for subjective physical symptoms related to URTS except nasal symptoms between the two groups during the intervention period; Table S1: Cumulative number of days with special notes; Table S2: Scores for subjective physical symptoms associated with URTS, with the exception of nasal symptoms, at week 1 and week 8; Table S3: POMS 2 T-scores at week 1 and week 8; Table S4: NK cell activity at week 1 and week 8.
